# Exploring Patients' Views of a Cognitive Behavioral Therapy-Based Website for the Self-Management of Irritable Bowel Syndrome Symptoms

**DOI:** 10.2196/jmir.2672

**Published:** 2013-09-03

**Authors:** Sarah Tonkin-Crine, Felicity L Bishop, Matthew Ellis, Rona Moss-Morris, Hazel Everitt

**Affiliations:** ^1^Primary Care and Population SciencesFaculty of MedicineUniversity of SouthamptonSouthamptonUnited Kingdom; ^2^Academic Unit of PsychologyFaculty of Social and Human SciencesUniversity of SouthamptonSouthamptonUnited Kingdom; ^3^Psychology DepartmentInstitute of PsychiatryKing’s College LondonLondonUnited Kingdom

**Keywords:** irritable bowel syndrome, cognitive behaviour therapy, Internet, primary care, qualitative research

## Abstract

**Background:**

Cognitive behavioral therapy (CBT) has been shown to have positive effects on the management of irritable bowel syndrome (IBS) symptoms. A factorial pilot randomized placebo-controlled trial (called MIBS) tested the potential effectiveness of a self-management CBT-based website alongside two medications: methylcellulose and mebeverine, and a placebo. The results showed no significant differences in quality of life or symptom severity measures, but enablement and participant’s global assessment of relief was higher in the website groups.

**Objective:**

To conduct a qualitative study nested within this trial, in order to explore patients’ views and experiences of using the CBT-based website to facilitate self-management of IBS.

**Methods:**

Semistructured interviews were carried out with patients who had used the website with one session of nurse support (n=16) or the website alone (n=15) while participating in the MIBS trial. An inductive thematic analysis was conducted.

**Results:**

We identified three types of engagement with the CBT-based website. One group of participants, mostly in the website-only condition, had limited or no engagement with the website. One group engaged with the content and advice on practical lifestyle changes. The final group of participants engaged with the content and advice on psychological aspects related to IBS. Similarities and differences between these three groups are explored.

**Conclusions:**

Teaching self-management techniques through a Web intervention was received positively by most of the participants. Concepts linked to cognitive aspects of CBT appeared to be harder for participants to engage with. Participants who received nurse support rated the cognitive aspects more positively, suggesting that some therapy support alongside the website should be considered. However, the Web format was preferred by some who favored anonymity as well as those who appreciated the accessibility and ease of use of this type of management. Suggestions on how to encourage engagement with Web interventions are discussed.

## Introduction

The prevalence of irritable bowel syndrome (IBS) is estimated to be 12% in northern European countries [1] with British organizations estimating that 10-22% of the UK population are affected [2]. NICE (National Institute for Health and Care Excellence) guidelines recommend that, in primary care, IBS patients be offered diet and lifestyle advice, pharmacological therapy, and psychological therapy [3]. Common medications offered to patients to help manage IBS are antispasmodics (eg, mebeverine) and bulking agents (eg, fybogel and methylcellulose). However, there is limited evidence about the effects of these medications [4]. Cognitive behavioral therapy (CBT), also identified in the NICE guidance, has shown to be helpful for patients with IBS; however, access to CBT is limited and the therapy can be expensive due to the costs of therapist training and the therapist time taken to deliver the CBT [3,5-7].

To improve accessibility to psychological therapies and reduce costs, research has investigated CBT Internet programs that help patients manage a wide variety of health conditions [8,9]. Reviews of this work indicate that Internet-based programs can be effective and suggest that Internet-based therapy can provide complementary and additional help for patients alongside existing health care [10].

Recent studies have started to investigate the effectiveness of Internet-delivered cognitive behavioral programs for patients with IBS [11-14]. Program content has included stress management and relaxation techniques [11], and mindfulness and exposure exercises [12], in addition to explaining the biopsychosocial model of IBS to participants. Studies have shown that Internet programs are effective at reducing experienced IBS symptoms and improving quality of life, compared to control conditions, that is, either a waiting list control [11] or a waiting list with access to an online discussion forum to try to control for nonspecific effects [12]. An additional follow-up indicated that patients could experience long-term beneficial effects on symptoms after participating in Internet programs [15], and a second study identified that CBT-based programs using exposure exercises may be a better option than programs that provide more general methods for reducing stress/symptoms [13]. However, a subsequent study investigating Internet-based CBT for IBS in a clinical population where patients were consecutively sampled as opposed to self-referral, reported smaller treatment effects than previous studies [14].

As part of the MIBS (Management of IBS) pilot trial, a Web-based CBT self-management program (named Regul8) was developed to help patients with IBS manage their symptoms [16,17]. This work followed the development of a paper-based CBT manual, which in conjunction with 3 hours of therapy time, was shown to be effective in reducing IBS symptom severity and impact [7]. The MIBS trial was designed to assess the effectiveness for IBS of the CBT-based self-management website and two commonly prescribed medications (mebeverine and methylcellulose) against placebo [17]. The results of the trial found no significant differences in IBS quality of life or symptom severity score between participants who received the website or medication at follow-up. However, enablement [18] and global assessment of relief [19] were significantly improved in participants who had received the website at 12 weeks compared to groups who had not received the website [17].

While research has suggested that Internet programs are effective at improving patients’ reported IBS symptoms, less is known about patients’ views and experiences of using such programs. Previous qualitative work has explored patients’ views of Internet-delivered CBT self-management programs to help management of depression [20-22]. These studies indicated that people may react differently to Internet interventions, some being able to work through programs alone, with others needing more encouragement and support to increase their motivation [20,21]. Being able to identify with the information provided and apply it to their own lives is also thought to be important for participants [20]. Last, the anonymity afforded by Internet-based programs may be attractive to participants who experience or anticipate stigma in face-to-face programs [20,22].

Inductive qualitative methods can help to explore an area where there is little existing knowledge. To date, there have been no reported qualitative investigations of people’s experiences of using an Internet-delivered CBT program for IBS. Following the MIBS pilot randomized control trial (RCT), we explored participants’ views of taking part in the trial, their experiences of using the Regul8 website, and their experiences of the trial medication. Our aim was to explore whether an Internet-delivered CBT program is acceptable to patients with IBS and, if so, to identify how it could be improved for use in future trials. While this study was carried out prior to having knowledge of the final trial results, the findings are presented with consideration to these.

## Methods

### The MIBS Trial and Website

This qualitative study was nested within the pilot MIBS RCT, which was designed to assess the potential effectiveness of two types of management strategies for symptoms of IBS [17]. The trial contained 9 participant groups (total N=135) in a 3x3 factorial design with variants of medication and the Web-based self-management program as the factors. Participants were asked to take 7 capsules a day for 6 weeks and either received one of two commonly used medications for IBS: mebeverine or methylcellulose, or a placebo. Participants were additionally randomized to three self-management groups: one group had access to the Regul8 website and a 30-45 minute telephone support session with a nurse to encourage engagement with the CBT program (n=46), a second group had access to the website only (n=45), and the third group had no website access (n=45).

**Figure 1:**
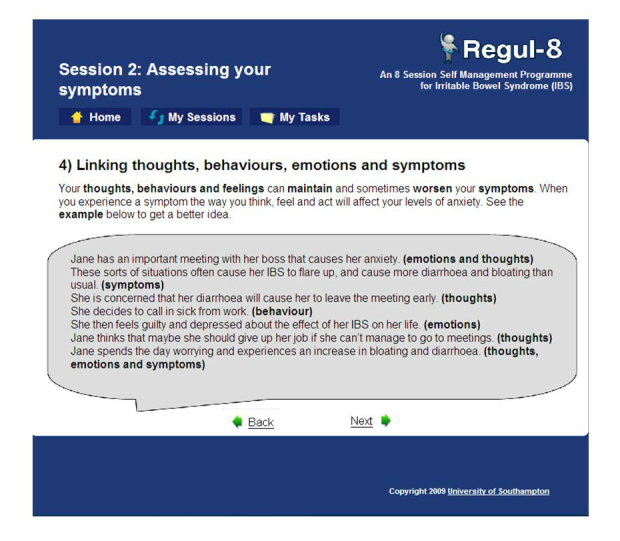
A MIBS webpage that gives participants an example of how thoughts, behaviors, emotions, and symptoms can interact.

The Regul8 website was designed to be interactive, and participants were provided with tasks to do within each session: these included recording symptoms and comparing them with stress and eating patterns, using goal-setting sheets for lifestyle change, and keeping thought records to identify and challenge unhelpful thoughts. Participants were able to use the Web program to keep track of goals or symptoms so that all their information was stored in one place and participants could discuss progress with these goals during the telephone support sessions where available. These interactive tasks allowed participants to focus on those parts of the program they felt were most relevant to them and to personalize the program, as a therapist may do. For example, participants may choose to have more goals related to changing their diet rather than using relaxation techniques. The website was split into 8 sessions that participants were required to complete over 6 weeks. The content of each session is described in Multimedia Appendix 1, and a webpage is shown in Figure 1. (MIBS pages are shown in Multimedia Appendix 2). More information on the program is available in the published protocol [16]. Separate “think aloud” [23] qualitative work was carried out during development of the website to inform the content and design in order for it to be understandable and user friendly for patients.

### Recruitment

**Figure 2:**
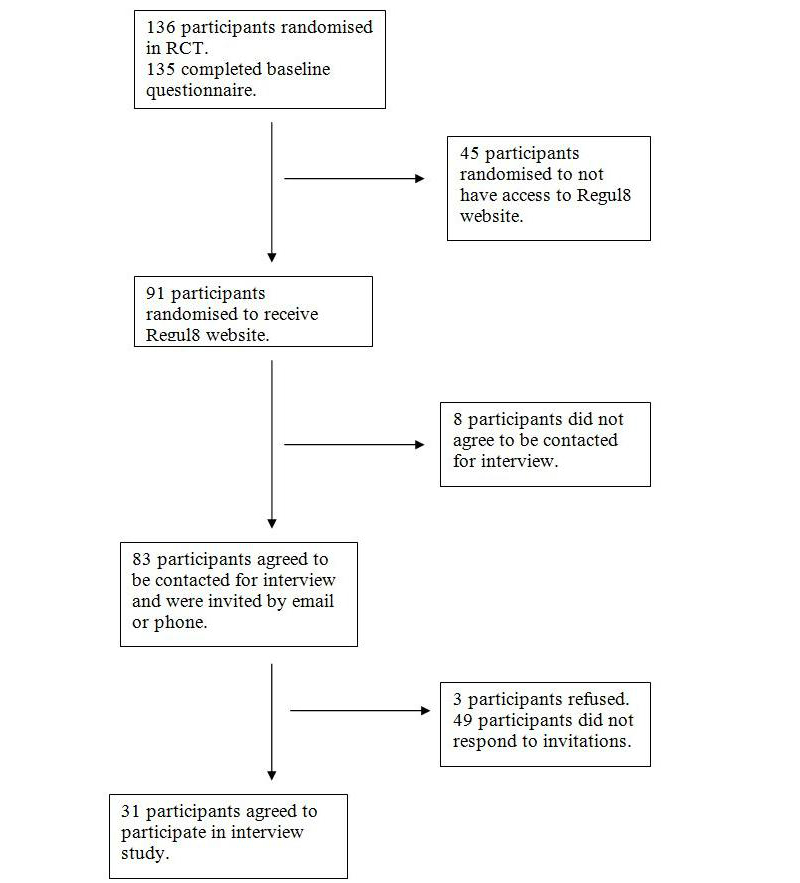
Flowchart showing how participants were identified from the original trial group.

All participants for this qualitative study were selected from the group of 91 who had taken part in the MIBS trial and who had had access to the Regul8 website (Figure 2). Participants were first contacted by email, and this was followed up by a phone call if there was no response within a week. Participants were called up to 3 times, at weekly intervals, if there was no response. Participants were purposively sampled to obtain participants of both genders, with a range of ages and with experience of using the website with and without nurse support. Participants were invited in the reverse order that they took part in the trial so that participants who had used the website most recently were contacted first. This was to recruit participants who had taken part in the trial most recently, with the assumption that these participants would better remember the content of the website and how they used it. Participants were interviewed when they had completed all aspects of the trial or when they were close to finishing the trial.

### Procedure

Telephone interviews were carried out by 2 researchers, STC and ME, between August 2010 and June 2011. Both interviewers were independent from the MIBS team and had had no involvement in the development of the website or management of the trial. Participants were made aware of this at the start of each interview to encourage interviewers to speak freely and honestly about their experiences of the MIBS trial. Questions focused on participants’ experience of taking part in the trial and their views and experiences of using the website and taking any type of medication for their symptoms. Questions related to the website asked about patients’ initial expectations of receiving a website as a management strategy for IBS, their feelings about receiving a website, what they liked and disliked about the website, and what they felt changed while working through the website. Questions were open-ended to allow participants to discuss anything they felt was relevant. All interviews were digitally recorded and transcribed verbatim. All identifiable information was deleted from transcripts. Interviews continued until researchers were satisfied that data had indicated saturation (no new themes identified in the data within the last few interviews) and when all participants who had volunteered to take part had been interviewed.

### Qualitative Analysis

Techniques from thematic analysis were used to analyze the data [24]. Thematic analysis was chosen to allow the researchers to take an inductive approach to analysis, ensuring that emerging themes were grounded in the original data and to help reduce the influence of the researchers’ existing knowledge and preconceptions. Transcripts were read and re-read to aid familiarity. One researcher, STC, coded the first ten transcripts, line-by-line, to develop initial codes. Initial codes were applied to subsequent transcripts and were refined and expanded. Codes were then compared for similarities and differences and grouped to form initial themes. NVivo 8 was used to facilitate coding. Initial themes were checked and approved by FB and HE. Themes were then compared across later transcripts, and new codes emerging from these were compared with earlier transcripts using a constant comparative technique [25]. Once all transcripts had been included in the analysis, code labels and titles of themes were checked and revised where appropriate with ongoing checks by FB and HE to ensure validity. Four themes were identified: (1) IBS is unpredictable and uncontrollable, (2) Perceptions of medication as a treatment for IBS, (3) Website format is acceptable, and (4) Engagement with the Regul8 website. This paper focuses on the fourth theme, Engagement with the Regul8 website. Within this theme, researchers identified three different ways that participants engaged with the Regul8 website. Three groups of participants were defined (by STC). Transcripts were examined independently by STC, HE, and FB to assess how transcripts fitted with these groups, and group definitions were then discussed and refined. A thematic map was developed to show the relationship between themes and the groups identified [24]. All transcripts were assigned to the final groups independently by the 3 researchers. Disagreements between assignments to the groups were identified and discussed, and transcripts were assigned when a consensus was reached.

## Results

### Participant Characteristics

Thirty-one participants were interviewed. Interviews lasted between 7 and 49 minutes, with a mean of 19 minutes. Those interviewed had a mean age of 51 years (SD 8.61), with ages ranging from 34-60 years; 60 was the maximum age to be eligible for inclusion in the MIBS trial.

The only significant differences between MIBS Regul8 participants who were interviewed and those that were not were gender and completion of 4 or more Web sessions (Table 1). As a group, interviewees completed one and a half more website sessions on average; however, this difference between groups was not statistically significant. Both groups had similar baseline symptom severity scores and quality of life scores. In both groups, participants were split equally between receiving the nurse support session or not.

### Findings

#### Website Format Is Acceptable

All participants reported that the website was well designed, easy to understand, and written in simple, nonspecialist language, for example, explaining it as “simple, concise and clear” (P2). However, all participants reported that the website took time to complete, and several mentioned that a user had to be self-motivated to work through the material:

I found it time consuming, you know, to log in, and I’m not one of those on the computer all day. I found it a bit time consuming and sometimes I must admit I thought I can’t be bothered to do this today and I didn’t but as I said, things had started to improve quite early on with the early part of the sessions.

P13

#### Engagement With the Regul8 Website

##### Overview

**Figure 3:**
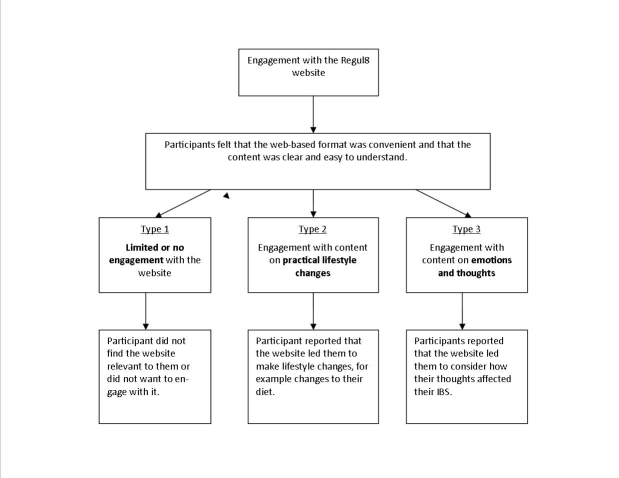
Thematic map identifying the 3 types of patient engagement with the Regul8 website.

Analysis revealed three types of engagement with the Regul8 website that emerged from participants’ discussions: (1) limited or no engagement, (2) engagement with content on how practical lifestyle changes may affect IBS, and (3) engagement with content on how emotions and thoughts may affect IBS (as well as practical lifestyle changes). Figure 3 summarizes these three types. Characteristics of each group appear in Table 2. Only 28 out of the 31 interviewees are represented in these groups since 3 participants (P26, P27, and P31) did not give enough detail on their use of the Regul8 website during their interviews.

**Table 1:** Characteristics of 31 participants who took part in interviews compared to the 60 participants who received access to the Regul8 website in the main trial.

Characteristics			31 participants interviewed	60 participants who refused or who did not take part in interviews	Significant differences between the two groups (*P*<.005)
**Demographics**					
	**Gender, n (%)**				*P*<.000
		Male	15 (49)	13 (22)	
		Female	16 (51)	47 (78)	
	**Age, years**				Not significant
		Mean average	51	43	
		SD	8.6	9.4	
		Range	34-61	22-60	
	**Ethnicity, n (%)**				Not significant
		White British	28 (91)	55 (95)	
		White other	2 (6)	3 (3)	
		Indian	0 (0)	1 (1)	
		Unknown	1 (3)	1 (1)	
					
**Baseline measures**					
	**IBS symptom severity score** ^a^ **(0-500)**				Not significant
		Mean average	241.6	237.6	
		Range	85-461	75-470	
	**IBS quality of life score** ^b^ **(0-100)**				Not significant
		Mean average	59.6	65.2	
		Range	18.38-88.97	20.59-96.32	
					
**Website group and compliance**					
	**Type of website access, n (%)**				Not significant
		Website with nurse support	16 (51)	30 (50)	
		Website only	15 (49)	30 (50)	
	**Compliance with website**				Not significant
		Mean average sessions completed	5.2 out of 8	3.7 out of 8	
	**Completed 4 or more website sessions, n (%)**				*P*<.001
		Yes (compliant)	21 (71)	30 (50)	
		No (non-compliant)	9 (29)	30 (50)	

^a^IBS symptom severity score indicates self-reported severity of symptoms by participants at baseline; higher scores represent more severe symptoms.

^b^IBS quality of life score indicates self-reported quality of life by participants at baseline; higher scores represent better quality of life.

**Table 2:** Participant characteristics for each of the three types of engagement identified.

Type	Number	Participant characteristics
Limited or no engagement with website	10	6 /10 compliant with the website. Completed on average 4.4 sessions out of 8. 2/10 participants received nurse telephone support
Engagement with content on practical lifestyle changes	6	4/6 compliant with the website. Completed on average 5.1 sessions out of 8. 4/6 received nurse telephone support
Engagement with content on emotions and thoughts	12	11/12 compliant with the website. Completed on average 6.4 out of 8. 8/12 received nurse telephone support.

##### Limited or No Engagement With the Regul8 Website

Ten participants reported either that they did not use the website at all or that they did not find it relevant and did not see it as a way to help them manage their IBS. Despite this, most participants in this group were recorded as completing 4 or more website sessions and therefore being compliant. As participants were able to click through website pages and select different sessions easily, it is possible that these participants browsed the majority of pages and were therefore recorded as having viewed the material, however, either did not absorb the information or decided it was irrelevant to them.

We identified three common obstacles that appeared to deter participants from engaging with the website. The first was an ongoing search for a “wonder drug”. Participants who were searching for a “magic pill” to “cure” their IBS resisted the idea that they might be able to (or would even want to) manage their illness through changing their thoughts and/or behaviors. They were hoping for a treatment that would enable them to “by and large lead a normal life” (P25) without disrupting their daily lives or taking time away from preferred or prioritized activities such as work and leisure. For example, in this extract one participant explains his desire for a medical treatment:

I don’t think I have much time, I think if I had time and I did some of the sort of more relaxation techniques at the time that the website was talking about them, then that would help, but to do some of these techniques I feel I need that, you know, almost one-on-one, you know, I think things like yoga could almost help it but I couldn’t just pick up a book and learn yoga, you know, it’s that kind of training I would love to do but um, time unfortunately doesn’t permit it and so I sort of put up with the symptoms, which is why I hoped it would be a wonder drug.

P5

Consequences of prioritizing a medical solution to IBS included seeing the website as unimportant within the trial and not seeing the website as a form of management in its own right. Indeed, 2 participants reported having made a conscious choice to abandon the website because they had stopped taking the trial medications.

Participants’ preconceptions about the impersonal nature of websites constituted a second obstacle to engagement. Some participants saw websites as a means to provide factual information and were expecting the website to provide extensive, authoritative, and new facts about IBS and its management. They were disappointed that the website did not meet their expectations: “it wasn’t extensive information, it was quite limited, I think I expected more” (P1). As described above, the website was interactive and did not merely provide information but required participants to self-assess, write down their own goals, or keep a written record of any unhelpful thoughts. This was difficult for some participants who believed that websites are unsuitable for interventions that require exploration of one’s personal thoughts and feelings about IBS. Regardless of their views on the legitimacy of a psychological approach to IBS in general, these participants did not feel that a website was the appropriate means of delivering such an intervention:

I just found the whole sort of process a little bit, um as I say a little bit sort of condescending, you know, to how a website, on an impersonal device like a computer, trying to give me a sort of counselling approach to managing the symptoms. [Interviewer: yes, yeah] so you know it was sort of, it was very impersonal [Interviewer: mm hmm] didn’t, didn’t gel with me at all.”

P5

Interestingly the majority of the participants who felt this way were in the group who did not receive the nurse telephone session to support their progress with the website (see Table 2).

The third obstacle to engagement related to poor perceived fit between the website and the individual and can be summarized by the phrase, “the website is not right for me”. Sometimes this centered on the psychological elements of the website, and obstacles to engaging with psychological content are discussed in detail below. Poor perceived fit was also related to perceived symptom severity and existing levels of knowledge. When they felt the website was not right for them, participants politely suggested other groups for whom it might be more suitable, including patients with more or less severe symptoms (with participants usually recommending the website for people who had different symptoms from themselves) or newly diagnosed patients who were assumed not to have an understanding of IBS and self-management techniques:

Well the trial wasn’t too bad, the website was ok, I think I would have put more effort into it if, like I say, I was having the issues with serious symptoms at the time but like I said I wasn’t really, so [there is] less motivation to work your way through the pages of the website.

P4

Despite not engaging with the interactive parts of the website, some participants nevertheless found it reassuring to have their existing beliefs about IBS confirmed and took comfort from feeling that they were not alone in their experiences of IBS. The latter might be a particularly valuable feature of websites for conditions such as IBS that patients often find difficult to discuss in everyday life:

I thought [the website] might have some suggestions as to things you could do to help relieve it. Which was sort of implicit there somewhere, so that was quite good. I think it was probably reassuring in a way…that obviously the symptoms I’d had were very common and were similar to other people...‘cause I hadn’t really talked to anyone else about it.

P9

##### Engagement With Content on Practical Lifestyle Changes

Six participants reported that the website had been helpful and had given them information and advice on making practical changes to their lifestyle:

I found [the website] actually really useful, the exercises…I have kind of tried to change slightly, and one of the bizarre things that was actually suggested, eat porridge…that made a big difference to settling my stomach down...there were some little things like that, that you went “oh I didn’t even realize”, never thought about it, so actually those things really helped.

P22

The changes participants reported were most often related to their diet but also included an increase in exercise, focusing on improving their sleep patterns, and practicing relaxation techniques. Sometimes participants reported that they had made significant changes that they felt were going to influence their IBS a great deal:

The exercise [section was useful]. I’m a child minder so I am sort of running around all day, but...it’s not sort of exercise, it’s not, you know, if I’m walking I’m walking with one of the kids so it’s not a walk that increases my heart rate... but you know we’ve been going up the park and that more and, you know, sort of running around the park more for more exercise. So [the website] was good.

P8

Other participants reported that the website had led them to make to smaller changes, which usually involved monitoring their behavior, but that had given them more of an insight into their IBS:

I looked at the action plans [on the website] and all it was really for me was just putting it in black and white, what I needed to do, which in some ways, it made you think a bit more, maybe took yourself out of the situation and made you think about things… more of an insight into what was going on, “Oh right, yeah, so that’s why that’s happened.” The one thing it did make me do was look at my diet more and made me associate different reactions and triggers for my IBS.

P11

As well as engaging with the content of the website, participants also commented on how they favored the interactive format of the Web-based program, which encouraged them to keep records of their thoughts and behaviors, and the nurse telephone support session, when available, and how these helped them to engage with the information.

[The nurse] made me feel perhaps better and realize that things perhaps had helped at the time that I hadn’t realized if you get what I mean? But that was not to do with the drugs, it was more with the [website] advice that was given, I found that more helpful.

P12

##### Engagement With Content on Psychological Aspects

Twelve participants spoke about how the website had led them to consider how their thoughts and emotions could affect their IBS:

[The website sessions] made you stop and think, “am I that kind of person that’s being described there?”…it’s all psychosomatic and I’m making it up in my head and I realized that it wasn’t necessarily that, because you have a physical symptom but then unfortunately because of the nature of IBS you’d…and the type of person you may be, the boom or bust person, that then unfortunately you may think “Oh my God, this is something wrong with me seriously” and then make the symptoms worse. Um, so I think having that actually on the Internet site it made me stop and reflect, and sometimes I think stop me from running ahead of myself.

P10

Several of the participants who had engaged with the psychological aspects mentioned how they had joined the trial because they were very eager to find something to help their IBS. Participants mentioned joining “out of desperation” because they had previously been “through hell” as a result of their IBS and because they felt they were “living on medication”. One participant specifically stated that she had joined the trial because of the “cognitive therapy” that was offered as part of the website.

All twelve participants reported that the website had helped to clarify how their thoughts and emotions may be connected to their symptoms. Some mentioned that the website had helped them identify how their emotions were connected to specific behaviors that they had subsequently tried to change:

I do find that if I’m stressed, het up or worried then I’m very, very likely to have an attack. I’m not, I don’t consciously think, “I’m going to have an attack now”...but whereas I got to the stage where I wouldn’t leave the house without making sure the medication was in my handbag and plenty of it...while I was doing the trial I never thought like that at all. I never, in fact, half the time I’d go out, and that was one of the things I had to do...was go out, try and put myself in a situation where I wouldn’t normally have gone without checking first I’d got it all, and put myself in those situations.

P7

Others talked about how the website had helped them understand the connection between their mind and body and how they were more comfortable considering how their thoughts and emotions may affect them:

I mean it’s not a disease, it’s an illness, you know it’s more than one thing and a lot of it is in the head and I think that makes you think about it, and um, think about your behavior and actions, and the consequences and your emotions and all that so yeah, I think that [the website] is very good.

P24

Approximately half of the participants, who had engaged with the psychological content of the website, also engaged with the advice on practical lifestyle changes.

As for group 2, this group also liked the nurse telephone support when it was available to them:

I think I got more out of it by having even the interim call with [nurse], during it, because um, the website can’t cater for everything without it being over-complex and pages and pages of options.

P2

Interestingly some felt that the website was a particularly good format for discussing a sensitive subject that some people may not want to talk about face-to-face:

[The website] explained [IBS] and it forced you to be quite open and honest probably more so than you would be in face to face conversation um, especially, um…you know it’s not the most pleasant of topics to discuss.

P2

We explored differences between participants who had engaged with the content on the psychological aspects of IBS and those who had not engaged with it. The majority of participants who did not engage with the psychological components of the website had only limited engagement with the website overall and seemed to miss out on the information on the influence of thoughts and emotions on IBS symptoms because of their lack of interaction with the website.

There were participants, however, who reported specific reasons for why they did not engage with the psychological content. These included participants who saw IBS as a physical and not psychological illness, participants who did not want to think about their IBS or any associated thoughts, those who did not understand how CBT and the tasks provided could help them, and those who felt negative thoughts were not relevant to them:

While I can accept that IBS might be brought on by stress which could be a symptom of what’s going on in your head, I couldn’t tie up that sort of logical, you know, write things down and try and feel better and that will make your symptoms better, I couldn’t make that link, I could see it was there but I just couldn’t do it and trying to do it just made me very frustrated.

P5

## Discussion

### Principal Findings

Participant feedback indicated that patients taking part in the MIBS trial engaged with the CBT self-management website to different extents that may help to explain the quantitative results of the MIBS trial [17]. While the majority of participants indicated that the website had been helpful to them and, as a result, that they had made subsequent changes to their lifestyle, a minority of those interviewed did not appear to gain any benefit from the website, either through improved symptoms, quality of life, or understanding their IBS better. Our analysis highlighted a variety of factors that may have shaped engagement with the website.

It was clear that medication was the only form of management that was seen as acceptable or potential efficacious by some interviewees. The trial design, investigating the Regul8 website alongside medication, meant that recruitment ran the risk of obtaining participants who were interested in only one type of intervention. Participants who had joined purely to try the medication may have had very little motivation to explore the website as a potential source of help. This is always a potential issue for recruitment to factorial trials and should be considered when providing trial information to potential participants and when providing trial materials once patients have been recruited.

Other participants who did not engage with the website reported that they found it too impersonal. This has been found in previous research that indicates that face-to-face delivery of psychological treatments is accepted by the majority but phone and Internet delivery is seen as less acceptable in comparison [26]. Of those interviewed, participants who had not received the nurse telephone support session generally reported being less engaged with the website. Input from a health care professional may have helped participants to relate the website information to their own lives, to understand the connection between their thoughts and symptoms, and to see the relevance of carrying out the tasks on the website. Previous studies have indicated that support given by a clinician or counsellor is associated with greater engagement with Web-based interventions, and interaction with health care professionals can significantly predict adherence to Web interventions [27-29]. Discussions with a professional may also have provided recognition and reassurance to participants who had previously had less constructive interactions with medical professionals. The level of support in this trial was minimal with one nurse telephone session of 30-45 minutes. This was much less support than offered within other trials of Web-based support for IBS that provided weekly feedback to participants [11,12]. It was encouraging to see that this support, while minimal, still had an impact on what participants reported as useful, with many saying that they valued this element of the program because it helped them to understand and follow the webpages.

Last, some participants reported not engaging with the website or with some specific website sections because they did not find it relevant to themselves. Some felt that the website was aimed at people who had less knowledge about IBS than themselves. The introduction section of the website, which gave lots of background knowledge on IBS, was a compulsory section that all participants were required to work through. Being presented with knowledge that was already very familiar may have decreased participants’ motivation to work through the remainder of the modules with some assuming that the website offered nothing new for them. Although the Regul8 website included tailoring by symptoms, some participants still felt that the website was aimed at people with different symptoms than themselves, either more or less serious. These assumptions could be addressed by changing the content of webpages, to emphasize the relevance to all patients or by increasing the ability of patients to skip information they already know. Participants who do not engage with certain sections of a Web intervention, for example the CBT aspects, may need further support and could benefit from one-on-one discussions with health care professionals as detailed above. It is important in this instance, however, to recognize that participants reported making changes to their lives and feeling benefits from following the website advice even though they had not engaged with the CBT aspects (eg, improved symptoms from changing their diet, using relaxation techniques to reduce anxiety related to IBS symptoms). This highlights the idea that engagement with CBT is not necessary in order to see positive effects on reported symptom control.

Overall, this study provided an insight into the views and experiences of patients with IBS symptoms in using a Web-based self-management program. Both the qualitative results presented here and the quantitative results of the trial [17] highlighted how the Regul8 website and design of the MIBS trial could be improved upon. The trial design can be improved by evaluating the Web intervention independently from any type of medication, which will prevent participants joining for access to medication only. This will also prevent any patient expectations that website content should mention medication. The delivery of the Regul8 website was acceptable to patients; however, the content could be improved by presenting optional units for patients with more information or allowing patients to skip sections, emphasizing that the website content is for everyone regardless of symptom severity, time since diagnosis, or prior knowledge, and by explaining that CBT techniques are useful for anyone regardless of whether an individual currently feels stressed or anxious. These lessons might also be applicable to trials of Web-based CBT in other chronic illnesses. A future larger RCT of the Regul8 website will further investigate the effectiveness of the website with telephone support on IBS symptoms and quality of life.

### Limitations

Patients taking part in the MIBS trial and these interviews were a self-selected group of volunteers and likely represented a population who were highly motivated to find a way to manage their IBS symptoms. This could result in participants being more positive about the management types available in the trial than the average IBS patient. It could also mean that patients were more motivated to use the website and therefore may have found it easier to work through.

Interviews are always likely to capture socially desirable responses. This was especially likely in these interviews as interviewees knew that interviews were being carried out in order to evaluate the trial and the website. Interviewers presented themselves as independent researchers, and we were encouraged to see that many participants seemed at ease when offering any negative feedback about the trial and/or website where relevant. This indicated that participants felt comfortable speaking openly and honestly about their experiences.

Due to the availability of independent interviewers, some interviews were carried out several months after participants had taken part in the study. As a result, some participants were unable to remember some aspects of the trial in order to comment on them in detail. Three interviews were not included in the analysis above because participants were not able to provide enough information on the Regul8 website. It was promising to see other participants’ experiences of the trial were, most often, positive and memorable and that they reported maintaining the changes they had made as a result of the website some months after completing the trial.

The qualitative results of this study were able to identify participants who reported that they had not used the website. This was despite quantitative results indicating that the same participants had been “compliant” with the website and had completed at least 4 of the 8 sessions. This discrepancy highlighted how quantitative measures gave a limited insight into participants’ use of the website and indicated that additional measures that recorded time spent on webpages and interaction with Web activities may provide more detailed information about Web use.

### Conclusions

Most patients reported gaining benefits, in the form of improved symptoms, greater quality of life, and/or greater understanding of IBS from following a CBT-based online self-management program either alone or with minimal nurse telephone support. Different levels of engagement with the website were seen across participants. For some, the website was helpful only for promoting lifestyle behavior change, while others also found benefit in following cognitive aspects such as challenging negative thoughts associated with IBS symptoms. Some participants may need extra support with the latter, and nurse or therapist input may help address patients’ preconceived ideas about the relevance of CBT or cognitions and emotions to their condition. Overall the Regul8 website offered an interactive self-management program that was well received by the majority of patients who found it relevant for their condition, and findings suggest further ways to improve the content of the intervention to encourage greater engagement.

## Multimedia Appendix 1

Summary of the Regul8 Web-based self-management sessions.

## Multimedia Appendix 2

A series of MIBS webpages showing how the website was tailored and personalized to individual needs.

## Abbreviations

CBT: cognitive behavioral therapy
IBS: irritable bowel syndrome
MIBS: management of IBS
NHS: National Health Service
NICE: National Institute of Health and Care Excellence
NIHR: National Institute of Health Research
RCT: randomized controlled trial
