# Supplementary material for: Exploring Patients' Views of a Cognitive Behavioral Therapy-Based Website for the Self-Management of Irritable Bowel Syndrome Symptoms
Source: J Med Internet Res. 2013 Sep 3;15(9):e190. doi: 10.2196/jmir.2672 (PMC3785978; doi:10.2196/jmir.2672)
Supplement: Supplementary file 1 [file jmir_v15i9e190_app1.pdf]

**Session 1:**<br><br>Understanding your IBS	Rationale for self-management which includes the following explanations:<br>1. Possible causes of IBS and illustrative physiology of the digestive system together with the functional changes that occur in the gut as a result of IBS.<br>2. How the autonomic nervous system ("fight-or-flight" stress system) may interact with the enteric nervous system.
**Session 2:**<br><br>Assessing your symptoms	Self-assessment of the interaction between thoughts, feeling and behaviors and how these can impact on stress levels and gut symptoms.<br><br>Development of a personal model of IBS which incorporates these elements.<br><br>Homework: Daily diaries of the severity and experience of IBS symptoms in conjunction with stress levels and eating routines/behaviors.
**Session 3.**<br><br>Managing Symptoms and Eating	Review of the symptom diary.<br><br>Behavioral management of the symptoms of diarrhoea and constipation, and common myths in this area are discussed. Goal setting is explained.<br><br>The importance of healthy, regular eating and not being overly focused on elimination is covered.<br><br>Homework: Goal setting for managing symptoms and regular/healthy eating. Goal setting, monitoring and evaluation continue weekly throughout the program.
**Session 4.**<br><br>Exercise and Activity	Importance of exercise in symptom management is covered.<br><br>Identifying activity patterns such as resting too much in response to symptoms or an all-or-nothing style of activity is addressed.<br><br>Homework: Goal setting for regular exercise and managing unhelpful activity patterns if relevant.
**Session 5.**<br><br>Identifying your thought patterns	Identifying unhelpful thought (negative automatic thoughts) in relation to high personal expectations and IBS symptoms is introduced.<br><br>Link between these thoughts, feelings, behaviors and symptoms is reinforced.<br><br>Homework: Goal setting plus daily thought records of unhelpful thoughts related to personal expectations and patterns of over activity.
**Session 6.**<br><br>Alternative thoughts	The steps for coming up with alternatives to unhelpful thoughts are covered together with personal examples.<br><br>Homework: Goal setting plus daily thought records including coming up with realistic alternative thoughts.
**Session 7.**<br><br>Managing Stress and Sleep	Basic stress management and sleep hygiene are discussed.<br><br>Diaphragmatic breathing, progressive muscle relaxation and guided imagery relaxation are presented in video and audio formats.

	Homework: Goal setting for stress management, relaxation techniques and good sleep habits.
**Session 8.** Managing flare-ups and the future	The probability of flare-ups is discussed and patients are encouraged to develop achievable, long term goals and to continue to employ the skills they have learnt throughout the manual to manage flare-ups and ongoing symptoms.
