# Supplementary material for: Exploring Patients' Views of a Cognitive Behavioral Therapy-Based Website for the Self-Management of Irritable Bowel Syndrome Symptoms
Source: J Med Internet Res. 2013 Sep 3;15(9):e190. doi: 10.2196/jmir.2672 (PMC3785978; doi:10.2196/jmir.2672)

## 5) Your Personal Model

**Let's begin looking at your behaviours.**

Below is a list of behaviours that might be familiar to you. If you do the behaviour, click the button. If none of these behaviours seem familiar you can write your own by selecting option 9.

Tips

**Note that you can only choose a maximum of three behaviours**

1. I skip meals when I have symptoms ☐
2. I avoid exercise when I have symptoms ☐
3. I avoid social events ☑
4. I check for toilets as soon as I arrive somewhere new ☐
5. I spend a long time on the toilet straining if I am constipated ☐
6. I check stools after I have been to the toilet ☐
7. I often change what I eat in response to my symptoms ☐
8. None of these behaviours apply to me ☐
9. I want to write my own behaviour ☐

Back Next

Copyright 2009 University of Southampton

## Session 2: Assessing your symptoms



### 5) Your Personal Model

Select any thoughts that are related to that behaviour or write your own. **Note that you can only choose a maximum of 2 thoughts**

| Behaviour | Associated thought | |
|---|---|---|
| 3. I avoid social events. | 1. If I go out I might have an accident. | ☐ |
| | 2. People might overhear me if I go to the toilet | ☐ |
| | 3. I might not know where the toilets are. | ☑ |
| | 4. I might pass wind in public. | ☐ |
| | 5. I might not be able to cope if my symptoms are bad when I am out. | ☐ |

**My own thoughts:** ________

Back    Next

Copyright 2009 University of Southampton

## 5) Your Personal Model

For your behaviour and thoughts, select any **emotions** that are related to that behaviour or write your own.

| Behaviour | Associated thought | Associated emotion | |
|---|---|---|---|
| 3. I avoid social events. | 3. I might not know where the toilets are. | 1. Depressed | ☑ |
| | | 2. Sad | ☑ |
| | | 3. Anxious | ☐ |
| | | 4. Embarrassed | ☑ |
| | | 5. Worried | ☐ |
| | | 6. Scared | ☐ |
| | | 7. Ashamed | ☐ |

**My own emotion:**

Back Next

Copyright 2009 University of Southampton

## 5) Your Personal Model

Here is your personal model showing the links between your behaviours, thoughts and emotions. We will focus on each of these over the course of the programme.

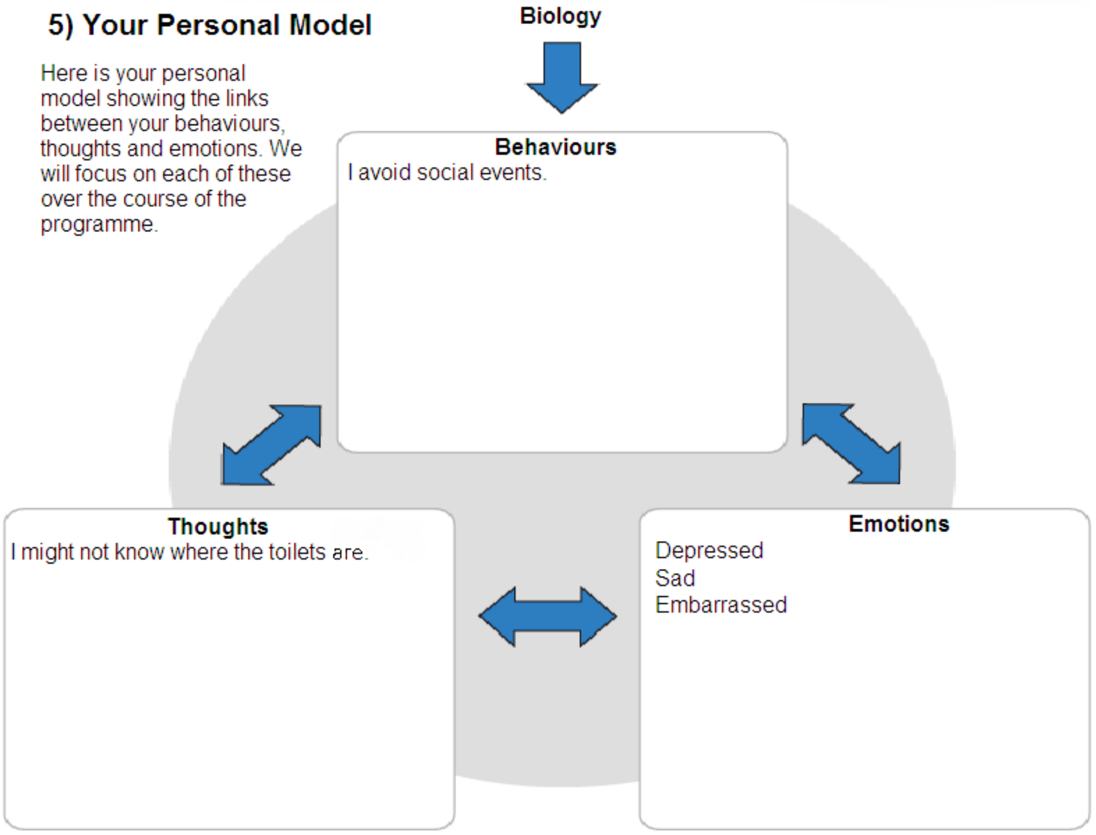

Supplement: Supplementary file 2 [file jmir_v15i9e190_app2.pdf]
